# Bax Inhibitor MoBi-1 Is Required for Conidiation, Pathogenicity, and Stress Responses in *Magnaporthe oryzae*

**DOI:** 10.3390/jof11050359

**Published:** 2025-05-05

**Authors:** Shuai Meng, Yangyang Shen, Dixuan Zhang, Liutao Bao, Hao Cao, Gening Song, Chenshun Xie, Jane S. Jagernath, Guoqiang Shen, Jie Chen

**Affiliations:** 1 National Joint Local Engineering Laboratory for High-Efficient Preparation of Biopesticide, College of Forestry and Biotechnology, Zhejiang Agriculture and Forestry University, Hangzhou 311300, China; mengshuai@zafu.edu.cn (S.M.); 18861609046@163.com (Y.S.); 19730560796@163.com (D.Z.); baoliutao@foxmail.com (L.B.); ch990805hao@163.com (H.C.); songgening@163.com (G.S.); 13588373373@163.com (C.X.); 2 Agricultural Department, Faculty of Technological Sciences, Anton de Kom University of Suriname, Leysweg 86, Building 7, Paramaribo 999182, Suriname; jane_jagernath31@hotmail.com; 3 Shaoxing Academy of Agricultural Sciences, Shaoxing 312000, China

**Keywords:** *Magnaporthe oryzae*, bax inhibitor-1, conidiation, stress responses, pathogenicity

## Abstract

*Magnaporthe oryzae* serves as a model organism for studying the molecular biology of filamentous fungi and the pathogenic mechanisms of fungal pathogens. It also poses a significant threat to rice production in China. Bax inhibitor-1 (Bi-1), a protein with evolutionary conservation, functions as an inhibitor of programmed cell death induced by the proapoptotic protein Bax. Despite the widespread presence of Bi-1 proteins in hyphal fungi, their biological functions have not been extensively characterized. Here, we characterized the function of *MoBI*-1, a putative Bax-inhibitor protein in *M. oryzae*, which is located in the mitochondria and participates in conidiation, stress adaptation, and pathogenicity. Further investigations revealed that MoBi-1 is also essential for the regulation of mitochondrial energy metabolism. Remarkably, experimental evidence indicates that MoBi-1 does not seem to function in inhibiting Bax-induced programmed cell death, thus lacking inherent Bax inhibitory function, which broadens the existing understanding of Bax inhibitor-1’s function and provides significant new insights into the disease-causing mechanisms of *M. oryzae*.

## 1. Introduction

Rice blast, caused by the filamentous ascomycete *Magnaporthe oryzae* (the rice blast fungus), is a highly destructive disease of rice that poses a significant threat to global rice production [1,2]. Mitochondria, as essential organelles in eukaryotic cells, not only provide energy but also participate in vital cellular activities such as cell signaling, differentiation, senescence, and death [3,4]. Recent research has revealed that mitochondria play a crucial role in fungal development and virulence, and mitophagy is a critical process in the pathogenicity of *M. oryzae* [5,6,7,8,9,10].

Bax inhibitor-1 (Bi-1) is a highly conserved multifunctional protein during evolution, primarily found in plants, fungi, bacteria, and viruses. The *BI-1* gene can attenuate or suppress stress-induced physiological cell death and plays a critical regulatory role in the interaction between programmed cell death and external stress [11]. Bi-1 was initially identified in yeast as a homolog of a human protein that inhibits Bax-mediated cell death processes [12]. The Bi-1 protein contains multiple transmembrane domains, is predominantly localized to the endoplasmic reticulum (ER), and acts as a resident sensor of the ER. In the mitochondrial pathway, Bi-1 inhibits the translocation of Bax from the cytosol to the mitochondria. Since Bi-1 interacts with the BH4 domain, it cannot directly bind to Bax or Bak. Instead, it modulates the ratio between Bi-1/Bcl-2/Bcl-XL and Bax by interacting with Bcl-2 and Bcl-XL, thereby influencing apoptosis [13,14]. Bi-1 possesses ion channel activity. In the endoplasmic reticulum (ER) pathway, cytosolic Ca^2^⁺ can leak extracellularly through this channel, triggering stress responses that induce apoptosis. Thus, Bi-1 suppresses mammalian cell apoptosis by regulating Ca^2^⁺ homeostasis [15]. When stress activates apoptosis-related signals on the ER, it also activates Bax, thereby linking these two pathways. The Bax inhibitor-1 (Bi-1) family consists of compact transmembrane proteins with significant anti-apoptotic activity. These evolutionarily conserved proteins are widely distributed across species and play key roles in regulating programmed cell death, cellular homeostasis, and stress responses [16,17].

In *Arabidopsis*, overexpression of *AtBI-1* suppresses Bax, H_2_O_2_, and salicylic acid-induced plant cell death and delays methyl jasmonate-induced leaf senescence by inhibiting MAP6 activation [18]. In *Nicotiana benthamiana*, silencing of *NbBI-1* reduces autophagy activity induced by N gene-mediated resistance to tobacco mosaic virus (TMV) and methyl viologen (MV), whereas overexpression of *NbBI-1* enhances autophagic activity, triggering autophagy-dependent cell death [19]. The wheat *TaBI-1* gene plays a critical role in plant resistance to salt and heat stress [20]. Ectopic expression of cotton *GhBI-1A* and *GhBI-1B* genes in *Arabidopsis* significantly improves tolerance to salt and tunicamycin [21]. In *Metarhizium robertsii*, the *MrBI-1* knockout mutant exhibits increased sensitivity to heat shock and is essential for virulence [11]. Silencing of *Ss-BI1* in *Sclerotinia sclerotiorum* reduces its pathogenicity, thermotolerance, and ER stress tolerance. In *Ustilaginoidea virens*, *UvBI-1* regulates vegetative growth, conidia production, stress tolerance, rice coleoptile elongation, and pathogenicity [22]. However, the biological functions and mechanisms of Bi-1 in *M. oryzae* remain largely unclear.

In this study, we demonstrated that MoBi-1 encodes a Bax inhibitor-1 (Bi-1) protein that is localized in the mitochondria and plays roles in conidiation, stress adaptation, and pathogenicity in *M. oryzae*. We also found that MoBi-1 plays an important role in mitochondrial energy metabolism. Additionally, our study indicates that MoBi-1 may not take part in suppressing the Bax-triggered cell death, and therefore have no function as a Bax inhibitor.

## 2. Materials and Methods

### 2.1. Sequence Analysis

All gene and protein sequences used in this study were downloaded from the National Center for Biotechnology Information (NCBI, https://www.ncbi.nlm.nih.gov/ 11 June 2021). Protein sequence alignments were performed using BioEdit, and the phylogenetic analyses were conducted using the MEGA 7.0 software [23].

### 2.2. Fungal Strains and Culture Media

The *M. oryzae* strain B157 was used as the wild type in this study. All the strains were cultured on complete medium (CM), basal medium (BM), basal medium without NH_4_NO_3_ (BM-N), and basal medium without glucose (BM-G) agar plates under 16 h of light and 8 h of dark at 25 °C [24]. Colony diameters were measured on CM plates at 7 days old, and the conidia were collected from 7-day-old colonies cultured on CM plates for testing. Three separate biological experiments were performed with three replicates at a time. The data were subjected to Duncan’s multiple range test.

### 2.3. Construction of the ΔMoBI-1 Strains and Complementation Analyses

To construct the *MoBI*-1 deletion vector, the 985 bp upstream and 1125 bp downstream flanking fragments of *MoBI*-1 were amplified from the genomic DNA of the B157 strain using the primer pairs MoBI-5F/R and MoBI-3F/R (Table S1). Then, these flanking fragments were ligated into *Hind* III/*Hind* III and *Xho*I/*BamH*I sites of pFGL821 (Addgene: 58224, www.addgene.org 29 June 2021) [25]. For the complementation, a genomic sequence driven by the *MoBI*-1 native promoter and 3′ UTR region was amplified and inserted into *EcoR*I/*Kpn*I-digested pFGL822 (Addgene: 58225, www.addgene.org 2 August 2021) with a glufosinate ammonium resistance gene. The complemented construct was transformed into the Δ*Mobi*-1 strain. Agrobacterium-mediated transformation (ATMT) was applied for genetic transformation in *M. oryzae*. The correct transformants of Δ*MoBI*-1 and the complementation assay were ascertained using Southern blot and qRT PCR analyses.

### 2.4. Southern Blotting, RNA Isolation, and qRT-PCR Analyses

The genomic DNA from the WT and mutant strains was extracted and digested with *Xba*I. Then, the digested products of genomic DNA were separated on a 0.8% agarose gel and mounted onto a positively charged nylon membrane (GE Healthcare, London, UK). The *MoBI*-1 gene probe was amplified from B157 genomic DNA using the primers MoBI-probeF/R (Table S1). The purified probe MoBI was subsequently DIG-labeled with Labeling Reagents (GE Healthcare, London, UK) to hybridize with the digested DNA products from the WT and Δ*MoBI*-1 mutant strains. The hybridization procedure was carried out according to the manufacturer’s instructions for the Amersham^TM^ AlkPhos Direct Labeling Reagents (GE Healthcare, London, UK). The signals of Southern blotting were detected by a ChemiDoc XRS+ system (Bio-Rad, Hercules, CA, USA).

Total RNA was extracted using the Fungal RNA Kit 200 (OMEGA, Norcross, GA, USA) according to the manufacturer’s instructions from the mycelia of WT and Δ*Mobi*-1 cultured in liquid CM. The cDNA of the first strand was synthesized by a reverse transcription kit (TaKaRa, Ōsaka shi, Japan) and analyzed by qRT-PCR using the TB Green^TM^ Premix Ex Taq^TM^ (Tli RnaseH Plus) (TaKaRa, Japan, Ōsaka shi).The *β-tubulin* gene was used as the endogenous reference gene, and the expression levels of MoBI-1, ATP-6, ATP-8, and ATP-9 were calculated using the 2^−∆∆Ct^ method [26]. The primers used are listed in Table S1.

### 2.5. Pathogenicity and Rice Infection Assay

For plant infection assays, the rice cultivar *Oryza sativa* cv. CO39, grown for three weeks, was used for the virulence test. Conidia harvested from B157, Δ*Mobi*-1, and Δ*Mobi*-1-C were suspended in a 0.1% (*w*/*v*) gelatin solution (1 × 10^5^ conidia/mL), then two milliliters of each solution was sprayed onto the plants. The disease lesions were photographed after 5 days post-inoculation under full humidity conditions at 25 °C. The infection process assay was conducted on a rice sheath cell. A rice sheath 5 cm in length was cut and dropped with about 50 μL of conidial suspension (1 × 10^5^ conidia/mL). After incubation for 12–48 h in a sealed chamber, a single layer of sheath cell was shaved to observe the invasive hyphae growing in the rice cell.

### 2.6. Subcellular Localization

For the fluorescent microscopic observation, the *MoBI*-1 gene with a native promoter and an open reading frame (ORF) was cloned with primers and ligated into the pFGL822-GFP [27] digested by *EcoR*I/*Kpn*I sites. The subsequent vector MoBi-1-mCherry was transformed into the Mito-GFP-expressing strain to produce coexpression transformants. Rescued transformants with GFP and mCherry signals were observed and documented by confocal fluorescent LSM700 microscope (Carl Zeiss Inc., Oberkochen, Germany). GFP and mCherry were imaged with 488 and 543 nm laser excitation, respectively. Images were conducted with ImageJ v 1.8.0 and Photoshop CS6 software.

### 2.7. Agrobacterium-Mediated Transient Expression of Proteins in N. benthamiana

The *MoBI*-1 coding region was cloned and ligated into a *Cla* I/*Sma* I-digested pGR107 vector [28]. The positive transformants were confirmed by sequencing and introduced into *A. tumefaciens* strain GV3101 by electroporation. The overnight-cultured *Agrobacterium* was collected and resuspended in 10 mM MgCl_2_ buffer. The final cell density was adjusted to an OD_600_ of 0.4. The *A. tumefaciens* containing the corresponding plasmid was infiltrated into 4–5-week-old *N. benthamiana* leaves using syringes without a needle. Photos were taken after 2–3 days post-inoculation.

## 3. Results

### 3.1. Identification and Knockout of MoBI-1 Gene in M. oryzae

**Figure 1:**
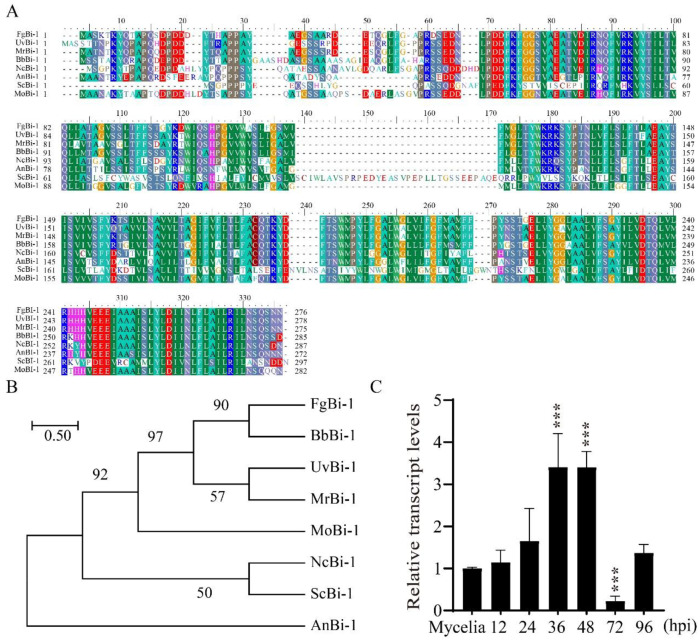
Phylogenetic tree depicting the relationship between putative orthologs of Bi-1 across different species. (**A**) The sequences included *M. oryzae* (*XP_003718554.1*), *Neurospora crassa* (*XP_011392998*), *Fusarium graminearum* (*XP_011317142*), *B. bassiana* (*XP_PMB66544.1*), *Metarhizium robertsii* (*XP_007826493.1*), *U. virens* (*KDB16044.1*), and *S. cerevisiae* (*KKZV10161.1*). Support was assessed using 100 rapid bootstraps. (**B**) Phylogenetic tree analysis of MoBi-1. The numbers at the branch are bootstrap values. The phylogenetic tree was constructed based on the alignment of the full sequences of Bi-1 from fungi by MEGA 7.0 using the neighbor-joining method. (**C**) The relative transcript abundance of the *MoBI-1* gene within *M. oryzae* was quantified. Utilizing the *β-tubulin* gene as a reference standard, the expression level of *MoBI-1* during the various infection phases was normalized against that observed during the vegetative growth phase. ***, *p* < 0.001.

To identify the homolog of Bi-1 in *M. oryzae*, the protein sequence of *S. cerevisiae* was used for a BLASTP search of *M. oryzae* in the NCBI database. The search results for *S. cerevisiae* Bi-1 identified the most closely matching protein, which is named MoBi-1 (XP_003720584.1) (Figure 1A). Sequence analysis using Motif Scan (http://smart.embl-heidelberg.de/, accessed on 20 October 2021) revealed that Bi-1 contained a conserved Bax inhibitor-1-like domain in fungi. A phylogenetic analysis of Bi-1 amino acid sequences from *Fusarium graminearum*, *Beauveria bassiana*, *U. virens*, *Metarhizium robertsii*, *Magnaporthe oryzae*, *Neurospora crassa*, *S. cerevisiae*, and *Aspergillus niger* showed that MoBi-1 is not closely related to Bi-1 homologs from other fungi (Figure 1B). Next, to investigate the potential role of MoBi-1, its transcript abundance was assessed via qRT-PCR across various infection stages of *M. oryzae*. Furthermore, an increase in MoBi-1 expression was observed during the infection process initiated by *M. oryzae* (Figure 1C). The results show that the transcriptional up-regulation of *MoBI-1* implicated a possible role of MoBi-1 during pathogenesis in *M. oryzae*.

**Figure 2:**
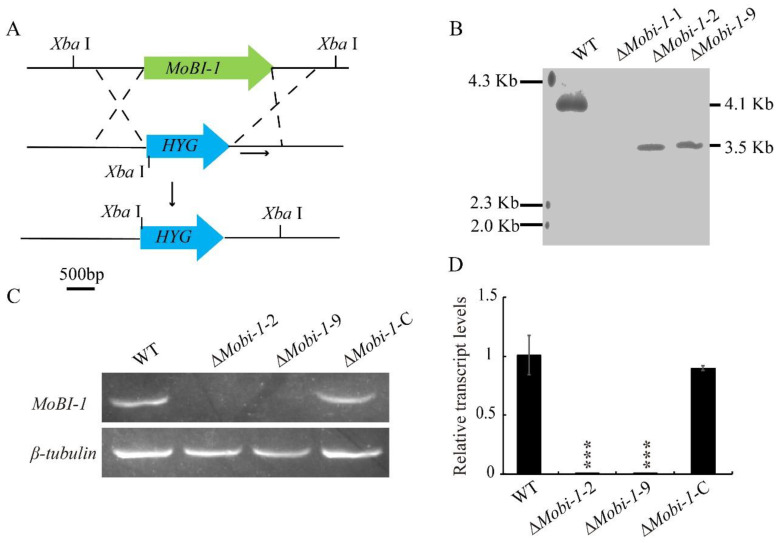
Methodologies for the generation of the *MoBI-1* gene disruption vector and Southern blotting. (**A**) Schematic representation of the gene deletion strategy. (**B**) Southern blot analysis of wild-type and Δ*Mobi-1* mutants. Genomic DNA isolated from the WT and Δ*Mobi-1* mutants were digested by *Xba* I and then hybridized with the probe, as shown in Figure 1A. (**C**) RT-PCR analysis of Δ*Mobi-1* mutant strains. (**D**) Quantitative real-time PCR (qRT-PCR) analysis of *MoBI-1* expression in the WT, Δ*Mobi-1* mutants, and complemented strain. Data are from three biological replicates. “***” asterisks indicate significant differences at a *p*-value < 0.001.

To investigate the biological roles of MoBi-1 in *M. oryzae*, knockout vectors carrying the *hygromycin resistant cassette* gene *HYG* were constructed and transferred into the wild-type strain (Figure 2A). Quantitative real-time PCR (qRT-PCR) and Southern blot assays were conducted to confirm the targeted gene knockout and exclude ectopic integrations (Figure 2B). The correct transformants were obtained with similar phenotypes, and Δ*Mobi*-1-2 and -9 were chosen for further experiments (Figure 2B). Complemented strains were generated by the full-length gene copy of *MoBI*-1 with its native promoter into the knockout mutant Δ*Mobi*-1-2 strain. The resultant *MoBI*-1-C strain was confirmed by PCR and qRT-PCR analyses, which showed that the abundance of *MoBI-1* transcript was comparable to that of the WT strain (Figure 2C,D).

### 3.2. The Role of MoBI-1 in Morphological Development

**Figure 3:**
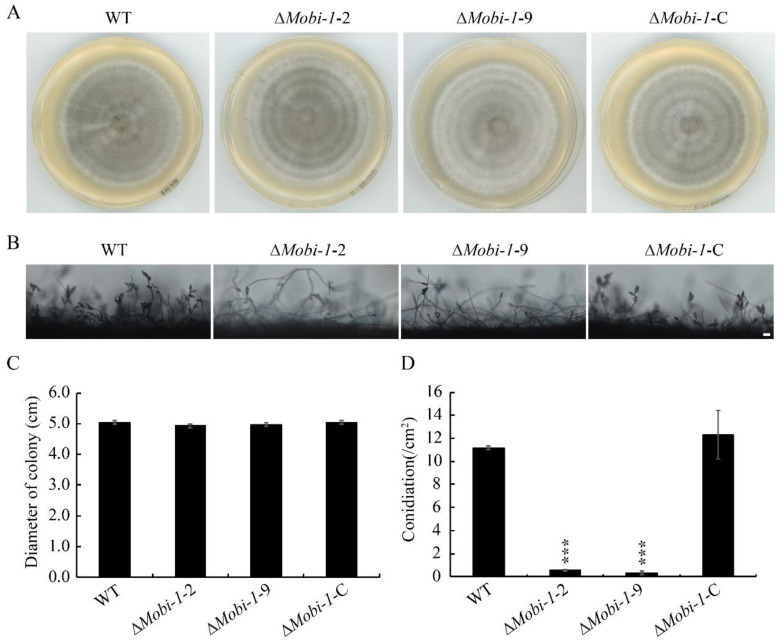
MoBi-1 is required for conidition in *M. oryzae*. (**A**) WT, Δ*Mobi-1*, and ΔMobi-1-C colonies, cultured at 28 °C on CM for seven days, were imaged. (**B**) Disruption of MoBI-1 resulted in conidition. Conidiophores were induced by incubating mycelial plugs under constant light for 24 h and examined by light microscope. Scale bar, 20 μm. (**C**) Colony diameter measurement of WT, MoBi-1 mutants, and complemented strain on CM medium. Colony diameters were measured after 7 days. The mutants of the ΔMobi-1 did not show significant phenotypic changes in growth characteristics on CM. (**D**) The average number of conidia of WT, ΔMobi-1 mutants, and the complementation strain on CM medium. Conidiation was measured by counting the number of conidia harvested from 7-day-old colonies at 25 °C. Error bars represent standard deviations. Three independent biological experiments were performed with three replicates each time and yielded similar results. The data were subjected to Duncan’s test, and the significant differences are shown in the figure with asterisks (***, *p*  <  0.001).

To investigate the roles of *MoBI-1* in fungal growth and development, the Δ*Mobi-1* mutants were cultured on complete medium (CM) agar plates after 6 days of incubation. In comparison with the WT and complementation strains, the colony diameter of Δ*Mobi*-1 mutant showed no significant differences (Figure 3A,C), indicating that MoBi-1 may not be involved in vegetative growth. However, the Δ*Mobi*-1 mutant produced almost no spores relative to the WT and Δ*Mobi-1*-C complementary strains (Figure 3B,D), suggesting that MoBi-1 plays an important role in the conidia of *M. oryzae*.

### 3.3. MoBI-1 Contributes to Full Virelence

**Figure 4:**
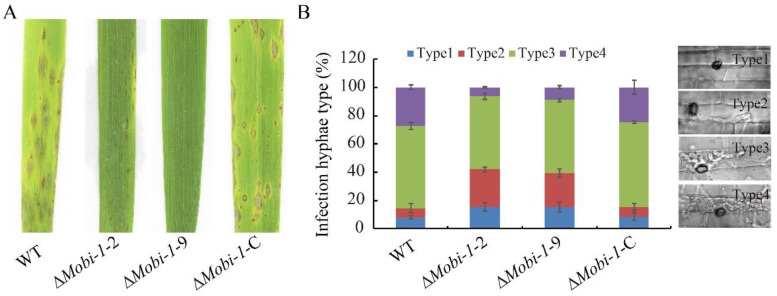
Effect of MoBi-1 on invasive hyphae growth and pathogenicity in *M. oryzae*. (**A**) Plant pathogenic assays. Deletion of MoBi-1 leads to reduced pathogenicity toward rice plants. Rice seedlings of the susceptible rice cultivar CO39 were sprayed with conidial suspensions (1 × 10^6^ spores/mL) of the WT strain and the Δ*Mobi-1* mutants and Δ*Mobi-1*-C. Leaves were photographed 7 d after inoculation. (**B**) Microscopic observation of penetration and infectious hyphae expansion on rice leaf-sheath cells. To determine the frequency of appressorium-mediated penetration, rice leaves were inoculated with conidial (1 × 10^6^ spores/mL) suspensions for 48 h. Each strain had at least 100 appressoria measured.

To determine whether *MoBI*-1 is required for pathogenicity virulence, conidial suspension (1 × 10^5^ conidia/mL) of WT, Δ*Mobi*-1-2, Δ*Mobi*-1-9, and Δ*Mobi-1*-C complementary strains were sprayed onto three-week-old rice leaves. The null mutant Δ*Mobi*-1-2 and Δ*Mobi*-1-9 showed few lesions on the rice leaves, while numerous typical spreading lesions were caused on the WT and complementation strain-infected rice leaves (Figure 4A). To elucidate the difference in the process of infection, we conducted an infection assay on the rice sheath to be certain of the early infection hyphae growth. Conidial suspension was dropped onto the rice sheath for 12–48 h in a sealed chamber [29]. At 48 hpi, invasive hyphae were observed and analyzed, with almost 60% of cells showing a type 4 lesion, nearly 30% displaying a type 3 lesion, and less than 10% showing type1 or type 2 lesions. However, only 10% of cells showed type 4 lesions, 60% of cells exhibited type 3 lesions, and 30% showed type 1 and type 2 lesions in the Δ*Mobi*-1 mutants (Figure 4B). These results indicated that MoBi-1 plays an important role in conidiation and pathogenicity in *M. oryzae*.

### 3.4. MoBi-1 Is Mainly Localized in the Mitochondria

**Figure 5:**
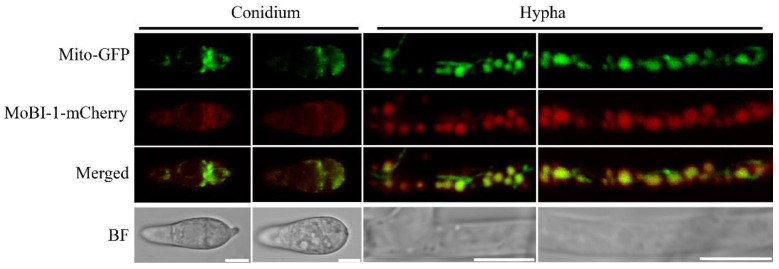
The MoBi-1 localizes to mitochondria. Localization of MoBi-1 protein in *M. oryzae*. Conidia and vegetative hyphae express the MoBi-1-mCherry fusion protein under confocal microscope. Conidia and vegetative hyphae express the MoBI-1-mCherry fusion protein under confocal microscope. Scale bar = 5 μm.

Numerous studies showed that Bax inhibitor-1 (Bi-1) is a highly conserved multifunctional protein in evolution, mainly in plants, fungi, bacteria, and viruses [11,15,30,31,32]. Thus, we found that MoBi-1 was mainly located in mitochondria and distinguished from other species in localization. A red fluorescent protein sequence was fused to the C-terminus of MoBi-1 using a native promoter, and the vector was introduced into the mitochondria marker Mito-GFP strain. The results indicated that MoBi-1-mCherry could co-localize with Mito-GFP in spores and mycelia (Figure 5).

### 3.5. MoBi-1 Cannot Inhibit BAX-Induced Cell Death in N. benthamiana

**Figure 6:**
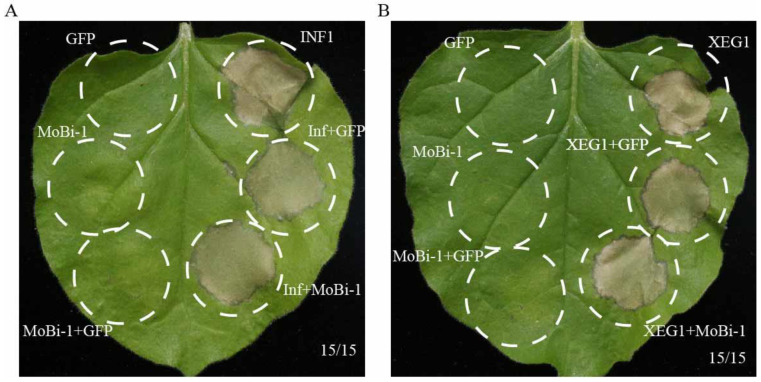
MoBi-1 could not suppress Bax-induced cell death. MoBI-1 did not inhibit INF1-induced (**A**) or XEG1-induced (**B**) cell death. GFP and MoBi-1 were expressed individually or with INF1 or XEG1 in *N. benthamiana* leaves. Cell death was observed and photographed after 7 days. At least three independent biological experiments were performed with 15 inoculated leaves each time.

Previous studies have reported that the Bi-1 domain has the function of inhibiting cell death induced by Bax [30,31,32,33,34,35]. Considering that MoBi-1 contains a Bi-1 domain, we ascertained whether *MoBI*-1 can also inhibit Bax-induced cell death. *Agrobacterium* strains carrying *MoBI*-1 and Bax were co-infiltrated into *N. benthaminan* leaves. MoBI-1, INF/XEG, and GFP were also expressed independently. The results showed MoBi-1 cannot inhibit the INF- or XEG-triggered cell death symptom in the infiltrated leaves (Figure 6). Furthermore, no cell death was detected when GFP and MoBi-1 separately were infiltrated into *N. benthamiana* leaves (Figure 6). These data indicated that MoBi-1 may not take part in suppressing the Bax triggered cell death, and therefore have no function as a Bax inhibitor.

### 3.6. Relative Expression of ATP Synthesis Genes Decreased

**Figure 7:**
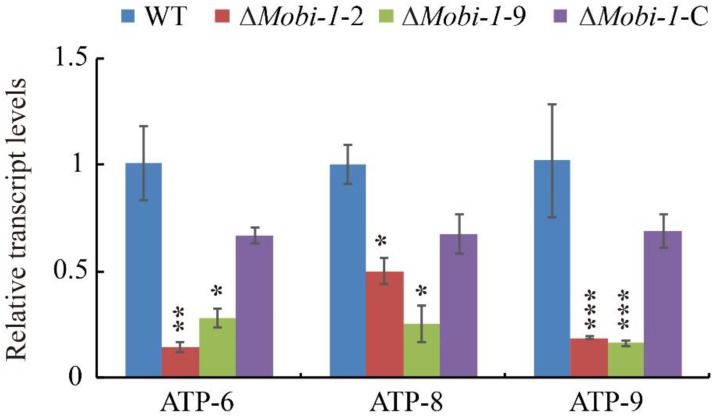
MoBi-1 is involved in the synthesis of mitochondrial energy metabolism. Differential ATP synthesis gene analysis on transcriptomes of the Δ*Mobi-1* mutants and those of the WT and complemented strain. There were consistent findings from three tests (* *p* < 0.01; ** *p* < 0.005; *** *p* < 0.001). Values are based on three biological samples, and error bars indicate the mean ± SD.

In all eukaryotic cells, the mitochondrion is a highly dynamic specialized organelle that serves as a master regulator of metabolism [36], and it is the main place of energy synthesis in the process of development [24]. Since the MoBi-1 protein localizes to the mitochondria, transcriptomes of the *MoBI*-1 mutants, as well as the WT and complemented strains, were analyzed for differential ATP synthesis genes, including mtATP6 (Genbank: MGG_21007), mtATP8 (Genbank: MGG_21008), and mtATP9 (Genbank: MGG_00892) [37,38]. The data showed that the expression levels of mtATP6, mtATP8, and mtATP9 were downregulated in Δ*Mobi*-1 mutant strains in comparison with the WT and Δ*Mobi-1*-C complementation strains (Figure 7). These results indicated that MoBi-1 plays an important role in the synthesis of mitochondrial energy metabolism.

### 3.7. MoBi-1 Is Involved in Responses to Nitrogen and Glucose

**Figure 8:**
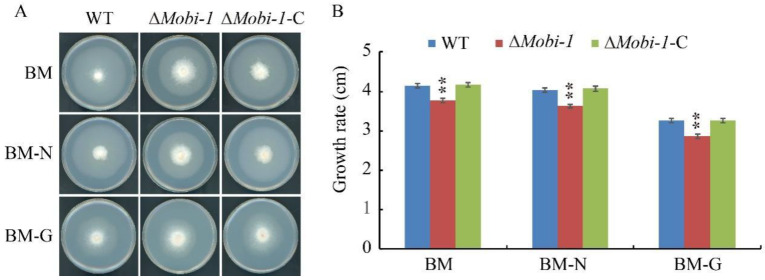
Determination of the sensitivity of the WT, Δ*Mobi-1*, and Δ*Mobi-1*-C strains to various stressors. (**A**) The representative strains were cultured on BM, BM-N, and BM-G plates at 25 °C for 7 days, and photographs were taken. (**B**) Colony diameter of the WT, Δ*Mobi-1* mutants, and complementation strains grown on BM, BM-N, and BM-G plates. The Δ*Mobi-1* mutant is compromised in terms of growth in basal medium. The data are presented as the average of 3 plates per treatment. Error bars indicate standard error. Duncan’s test was applied to the results, and the significant differences are shown in the figure with two asterisks (**, *p* < 0.05).

Carbon and nitrogen sources play an important role in fungal growth and development. To study the function of MoBi-1 in nitrogen and glucose on mycelia growth, the Δ*MoBI*-1 mutants, WT, and complementation strains were grown on BM medium, BM-N medium (BM medium without nitrogen source), and BM-G (BM medium without carbon source) for 7 d at 28 °C. The results showed that Δ*Moi*-1 mutants were not able to efficiently utilize the carbon and nitrogen sources and grew slower than the WT and Δ*Mobi-1*-C complementation strains (Figure 8), indicating that *Mo*Bi-1 is involved in glucose and nitrogen utilization.

## 4. Discussion

The rice blast fungus *M. oryzae* is a well-known model for fungal–plant interactions. Until now, a large number of mitochondria-related genes have been identified for functional analysis. They are primarily involved in the process of fungus development, physiology, and pathogenicity [6,8,38,39]. The rice blast fungus *Magnaporthe oryzae* is a highly specialized pathogen renowned for its exceptional morphogenetic and biochemical adaptations for a parasitic lifestyle, posing a significant threat to rice cultivation worldwide. Substantial progress has been made in understanding the molecular mechanisms underlying this destructive disease, and numerous genes crucial for the early stages of infection have been identified [40].

The mitochondrion, a highly specialized and dynamic organelle, resides within all eukaryotic cells and functions as a central regulator of cellular metabolism [36]. These organelles ensure an uninterrupted supply of adenosine triphosphate (ATP), the primary energy source for cellular activities [41]. Phytopathogenic fungi, organisms that cause diseases in plants, acquire the energy necessary for their metabolic processes from substrates obtained from living or recently deceased plant matter [42]. The endosymbiotic acquisition of mitochondria, a pivotal event in eukaryotic evolution, significantly enhanced the capacity for energy production and sophisticated metabolic regulation, laying the foundation for the emergence of complex life. Mitochondria, which are not only the primary energy generators in modern eukaryotic cells but are also integral players in a wide array of cellular functions, orchestrate these processes through oxidative phosphorylation [43]. The mitochondrial respiratory chain, a major source of reactive oxygen species (ROS), serves as a convergence point for numerous metabolic pathways [44].

Programmed cell death, an essential mechanism for eliminating unwanted cells, is a fundamental process inherent in all living organisms [45]. Apoptosis, the most prevalent form of this cellular demise, is a meticulously regulated process that selectively removes cells during various biological events [40]. The Bax inhibitor-1 (Bi-1) protein family, a group of small transmembrane proteins, exhibits potent anti-apoptotic properties and is remarkably conserved across diverse organisms [30]. Recent studies have unveiled intricate interactions between Bi-1 and Bcl-2 protein families, demonstrating that BI-1 proteins can either amplify the anti-apoptotic activity of Bcl-2 or counteract cell death triggered by the pro-apoptotic protein Bax. Moreover, Bi-1 plays a crucial role in mediating cellular responses to both biotic and abiotic stresses in a wide range of organisms, including animals, plants, fungi, and yeast [30,46,47].

While BI-1 proteins are ubiquitous within filamentous fungi, functional characterization remains limited for most. In *U. virens*, UvBi-1 exerts a suppressive influence on mycelial growth and conidiation, and is indispensable for stress tolerance, cell wall integrity, the production of secondary metabolites, and the ability to cause disease [22]. In *M. robertsii*, a fungal pathogen, the absence of MrBi-1 resulted in impaired fungal development, attenuated virulence, and compromised heat tolerance. Inactivation of MrBi-1 diminished fungal resistance to farnesol but had no discernible effect on hydrogen peroxide resistance, suggesting that MrBi-1 contributes to apoptosis-like cell death through the endoplasmic reticulum stress signaling pathway rather than the more conventional mitochondria-dependent pathway [11]. The potential of apoptotic proteins as targets for antifungal drug development has been recognized. However, the successful implementation of this approach hinges upon a more comprehensive understanding of fungal apoptotic networks and the identification of key regulatory proteins involved in apoptosis-like cell death within these organisms.

In conclusion, this study provides evidence that the Bax inhibitor-1 family MoBi-1 encodes the putative Bax-inhibitor protein, which plays an important role in sporulation and the full virulence of *M. oryzae*. Our findings further reveal that MoBi-1 serves a critical regulatory role in mitochondrial energy metabolism biosynthesis. Given the localization of MoBi-1 within mitochondria and its role in ATP production, the potential involvement of MoBi-1 in the mitochondrial autophagy pathway warrants additional investigation. Notably, the experimental evidence suggests that MoBi-1 does not appear to participate in the suppression of Bax-mediated apoptotic pathways, thereby lacking intrinsic activity as a Bax inhibitor. These results extend the current understanding of the function of Bax inhibitor-1 and provide novel and important insights into the pathogenic mechanisms of *M. oryzae*.

## Data Availability

The data presented in this study are available in the article and in its online Supplementary Material.

## Supplementary Materials

The following supporting information can be downloaded at: https://www.mdpi.com/article/10.3390/jof11050359/s1, Table S1: Primers used in this study.
