# Supplementary material for: Bax Inhibitor MoBi-1 Is Required for Conidiation, Pathogenicity, and Stress Responses in Magnaporthe oryzae
Source: J Fungi (Basel). 2025 May 5;11(5):359. doi: 10.3390/jof11050359 (PMC12112661; doi:10.3390/jof11050359)
Supplement: Supplementary file 1 [file jof-11-00359-s001.zip › jof-3580230-supplementary.pdf]

Table S1. Primers used in this study.

**Primer**	**Sequence (5'-3')**	**Application**
MoBI-1-5F	attattatggagaaactcgagGCCTACACACGGATAGCGAGA	Amplifying 5' flank sequence of *MoBI-1* for
MoBI-1-5R	gactctagaactagtggatccGGTAGGCAGACAAGGCAGAGAA	gene deletion
MoBI-1-3F	gaattgcatgtcgacctgcagGGCGACGAGTAGTTCGGTTTC	Amplifying 3' flank sequence of *MoBI-1* for
MoBI-1-3R	acgacggccagtgccaagcttTCTAACGCTGGTAACGCTGGT	gene deletion
Hyg-5R	CGCGTGCCTAGACTTACACT	Transformants screening
MoBI-1-cf	tatggagaaactcgagaattcTGATACCAACGCTTTGTGAATGT	
MoBI-1-cr	ctagtggatcccccgggtaccCAGCCACCACTTGAGGGATTT	Construction of complementation vector
MoBI-1-mCF	AGAAACTCGAGAATTCGCGAAATTGGCCGAGTCTTG	
MoBI-1-mCR	TCGCCCTTGCTCACGGTACCATTCTGCTGCTGGCTGTTAA	Construction of fluorescent localization
MoBI-1RT-F	GAGCGCACCATCTTCTTCAA	vector
MoBI-1RT-R	TCCTTGAAGTCGATGCCCTT	Analysis the expression level of *MoBI-1*
ACTIN-F	ACAACTTCGTCTTCGGTCAG	gene of *M. oryzae*
ACTIN-R	GTGATCTGGAAACCCTGGAG	Analysis the expression level of *ACTIN* gene
MoBI-1-probeF	gaattgcatgtcgacctgcagGGCGACGAGTAGTTCGGTTTC	of *M. oryzae*
MoBI-1-probeR	acgacggccagtgccaagcttTCTAACGCTGGTAACGCTGGT	Amplification of probe for Southern blot
		assay
